# The Future of the Medical Profession

**Published:** 1918-04-27

**Authors:** 


The Hospital

The Workers' Newspaper of Administrative Medicine and Institutional Life, Administration, National Insurance and Health.

No. 1664, Vol. LXIV. SATURDAY, APRIL 27, 1918. PRICE ONE PENNY

### THE FUTURE OF THE MEDICAL PROFESSION.

The recent discussion at the Royal Society of Medicine on the future of the medical profession reveals some of the movements of opinion which are being agitated within professional circles and in view of the developments which may reasonably be expected as results, more or less direct, of the present war. As all the speakers were medical practitioners the discussion has a certain domestic quality, though the consideration of the public interest received full justice. All the same it has to be remembered that where the public health and public funds are concerned the issues cannot be settled merely by professional opinion. Legislators and Government authorities will necessarily have their say, and indeed it is with them that there remains the last word. Yet if the doctors are united, and if they are led wisely and courageously, they must, in a large measure, command the situation, and hence the discussion in question has considerable significance. Where it fails is in not betraying what the general practitioner thinks of the position, as the speeches were made almost entirely by official persons or by doctors not engaged in general practice. But some of the speakers may be regarded as exercising a greater or less measure of influence on their colleagues, and at all events the views announced may all claim some degree of professional support.

In certain respects the speakers were substantially of one and the same opinion. All were agreed that changes are needed, and that changes are inevitable. They agreed, too, that these changes would cost money, and, as is not uncommon in such circumstances, there was a unanimous conclusion that the money must be provided by public funds. The justification for this conclusion was found in the contention that the new developments would all have as their motive and influence the promotion of the health of the nation, and the claim, it will be allowed, is a valid one. Laboratory examinations and investigations by means of specialised apparatus are expensive undertakings, and they have become an essential part of the equipment of modern medicine. If, as more than one speaker urged, the opportunities for effective treatment ought to be as fully at the disposal of the poor as of the rich, it is certain that money will be needed, and no source of supply offers itself outside the taxpayer. A point of importance to be noted in this respect is the admission that under the National Insurance scheme the patients are far from possessing anything like full facilities for modern treatment, and this supports what we have often affirmed—namely, that but for the services of our voluntary hospitals this scheme would long since have broken down. Everyone will sympathise with the ideal that every citizen should be able to command the most effective medical treatment known to science, but let it be well remembered that such an undertaking will mean a lot of money. The money may be a most excellent national investment, but it will have to be forthcoming. It is common ground that anything like equal opportunities do not exist now, and that panel practice as at present organised does not provide them. If they are to be supplied in the public interest it is from public sources that the funds must come. From this conclusion there follows another step—namely, that there must be public control, or some measure of public control, of medical practice. Subsidies and independence are mutually incompatible. An argument which urges that hospitals and private practice must both be State-aided, and at the same time suggests the maintenance of the present independent relations of the practitioner, found a voice in the discussion, but it is impossible to regard it as secure standing ground. The State has greater need than ever to be scrupulous in public expenditure and will certainly not consent to pay for more efficient medical treatment and yet to leave the administration of the funds in the hands of the medical profession. To hope for any such arrangement is a vain thing.

It is particularly to be noted that what we have just said of the discussion refers not merely to preventive medicine, but also to medical treatment. The former is already a public service with the charges and supervision which such a term implies. What is now recognised is that the actual treatment of sick people cannot be conducted at an efficient level without financial help from the State, assuming, that is, that such treatment is to be available for every citizen, whether he is or is not able to pay for it. To say, as responsible voices now say, that here also public funds must be available is a very large proposition. We are not in the least concerned to dispute it, but we desire to note that its acceptance means both a very large expenditure and also some degree of control over the activities of the medical practitioner.

The acute point of separation as to the future of the profession arises when the speakers come to deal with the position of the practitioner in relation to the State. All demand laboratories, $x$-rays, modern diagnostic methods, expert advice, expert treatment, and the help of nurses. But while one group claims that these must be supplied, its advocates will not have the profession organised as a branch of the Civil Service, but wish the practitioner to stand to the public in his present relation. The opposition regards such a position as impossible, and urges on its merits State organisation and control. Here is the sharp difference between the two groups, and it will be well for it thoroughly to be fought out.

